# Recent Progresses in Application of Membrane Bioreactors in Production of Biohydrogen

**DOI:** 10.3390/membranes9080100

**Published:** 2019-08-10

**Authors:** Bahman Jabbari, Elham Jalilnejad, Kamran Ghasemzadeh, Adolfo Iulianelli

**Affiliations:** 1 Faculty of Chemical Engineering, Urmia University of Technology, Urmia 57166-17165, Iran; 2 Institute on Membrane Technology of the Italian National Research Council (CNR-ITM), via P. Bucci Cubo 17/C, 87036 Rende (CS), Italy

**Keywords:** membrane bioreactor, biohydrogen, dark fermentation, anaerobic process, fouling, polymeric membranes, H_2_ separation

## Abstract

Biohydrogen is a clean and viable energy carrier generated through various green and renewable energy sources such as biomass. This review focused on the application of membrane bioreactors (MBRs), emphasizing the combination of these devices with biological processes, for bio-derived hydrogen production. Direct biophotolysis, indirect biophotolysis, photo-fermentation, dark fermentation, and conventional techniques are discussed as the common methods of biohydrogen production. The anaerobic process membrane bioreactors (AnMBRs) technology is presented and discussed as a preferable choice for producing biohydrogen due to its low cost and the ability of overcoming problems posed by carbon emissions. General features of AnMBRs and operational parameters are comprehensively overviewed. Although MBRs are being used as a well-established and mature technology with many full-scale plants around the world, membrane fouling still remains a serious obstacle and a future challenge. Therefore, this review highlights the main benefits and drawbacks of MBRs application, also discussing the comparison between organic and inorganic membranes utilization to determine which may constitute the best solution for providing pure hydrogen. Nevertheless, research is still needed to overcome remaining barriers to practical applications such as low yields and production rates, and to identify biohydrogen as one of the most appealing renewable energies in the future.

## 1. Introduction

Hydrogen is the simplest and the most abundant element on Earth, and it can be found in many sources such as water, hydrocarbon fuels, inorganic substances, etc. Hydrogen possesses the highest energy content per unit weight (142 kJ/g) and it represents a potential energy carrier due to its high energy density and low pollutant generation [1]. However, its conversion to usable power is highly efficient and environmental friendly, and produces water instead of greenhouse gases. Hydrogen is conventionally produced by steam reforming of natural gas, in which a non-renewable energy source is consumed for this purpose. Since non-renewable sources are depleting and are not sustainable for the long-term future, it seems necessary to explore sustainable and renewable energy sources such as biomass for biohydrogen production. Various biological processes using different hydrogen producing microorganisms in various bioreactors were studied and proposed in the specialized literature for biohydrogen production [2,3,4,5]. These processes are operated at ambient temperature and atmospheric pressure, requiring a lower amount of energy. Biohydrogen could be generated via both light-dependent and dark fermentative processes, even though there is remarkable progress toward the latter for an ongoing practice use [5,6,7]. In dark fermentation, substrates are converted by anaerobic bacteria grown in the dark. Hence, this technique is considered to be more feasible and cost effective than light-dependent processes, with net energy ratio of 1.9 [8,9].

As the main bioprocess occurs in the bioreactors, consequently their typology, configuration, and design significantly affect the efficiency of the biohydrogen production process itself.Continuous stirred tank reactors (CSTRs) are conventionally used for biohydrogen production in continuous mode, because of its simple configuration, easy functionality, effective homogeneous mixing, high mass transfer, and operation under variable conditions of the substrate, pH and hydraulic retention time (HRT) [10,11]. Nevertheless, biomass washout might occur in these reactors at short HRTs, resulting in low biohydrogen production rates. Coupling traditional anaerobic fermenters with membrane technology leads to the AnMBRs, which constitute an innovative technology in biohydrogen production. High biomass concentration, high conversion efficiency and less sludge production are some of the advantages of AnMBRs utilization over conventional CSTRs. Operating conditions of the fermentation process (e.g., pH, temperature, hydrogen partial pressure, mass transfer, substrate concentration, etc.) and also hydrogen purification methods are the most important parameters affecting the biohydrogen production efficiency. However, low yields and production rates are major barriers to the practical application of biohydrogen technologies [9].

In this review, particular attention is paid towards fermentative hydrogen production technologies and their improvements over the conventional techniques. General features of MBRs, especially AnMBRs, are discussed focusing on hydrogen production performance. A more thorough discussion is carried out with respect to factors affecting the biohydrogen production and techniques to improve its yields and generation rates. However, since membrane fouling still represents the main drawback for a wider MBRs application, membrane fouling types, mitigations and cleaning methods are here discussed and reviewed. Regarding the fact that the success of biohydrogen results to be dependent on the successfulness of the downstream purification technology, therefore, the proper membranes for biohydrogen purification are also presented along with essential separation conditions in the last section of this review.

## 2. Basic Biohydrogen Production Technologies

Hydrogen can be considered as a clean energy carrier similar to electricity. Since hydrogen is always combined with other elements, it cannot be found naturally on Earth and must be manufactured. Available resources that can be used for production of hydrogen are fossil fuels, biomass and water. It can be generated by various methods based on different sources of hydrogen, while the environmental impact and the energy efficiency of hydrogen generation depend on how it is produced. According to the literature, the most developed hydrogen production technologies are: water electrolysis, thermo-chemical hydrogen production (e.g., natural gas reforming/gasification, reforming of renewable feedstocks), and biological hydrogen production [6,12,13].

Biological processes for hydrogen production gained a lot of attention in the last decade since the primary challenge is constituted by the need of reducing the cost of production technologies. Biological hydrogen production technologies can be operated at ambient temperature and pressure with minimal energy consumption, resulting environmentally friendly processes [9]. However, low hydrogen yields and production rates are considered as the major constraints to the commercialization of these processes.

Biohydrogen production methods can be categorized into biophotolysis (direct and indirect), photo-fermentation and dark fermentation. Biophotolysis and photo-fermentation can also be classified as light-dependent processes, while dark fermentation is the major light independent process. Either of two classes of enzymes, the hydrogenases or the nitrogenases are involved in these processes for hydrogen evolution. These processes are discussed in detail in the following sections [4,14,15].

### 2.1. Fermentation

#### 2.1.1. Photo-Fermentation

In photo-fermentation, different photosynthetic bacteria are involved in a fermentative conversion process of different substrates. Since this process is light dependent, sunlight plays the main role as the source of energy which converts organic compounds into hydrogen and CO_2_ [16].

In the processes fermented by photosynthetic bacteria, molecular hydrogen is produced through catalyzing of organic compounds by nitrogenase under nitrogen-deficient conditions. These bacteria are capable of consuming organic acids like acetic acid as electron donors. Using adenosine triphosphate (ATP) energy, the released electrons could be transferred to the nitrogenase by ferredoxin. Extra ATP energy is consumed in this stage to reduce proton into hydrogen by nitrogenase in absence of nitrogen. The overall reactions of hydrogen production are reported below [17,18]:

C_6_H_12_O_6_ + 6H_2_O + light energy→ 12H_2_ + 6CO_2_, ΔG^0^ = +3.2 kJ (1)

The conversion of acetic acid into hydrogen and CO_2_ through a nitrogen-deficient fermentative process occurs as in Equation (2):

CH_3_COOH + 2H_2_O + light → 4H_2_ + 2CO_2_ (2)

As it is obvious, the organic acid could be oxidized using sun light as the energy source, and tricarboxylic acid cycle (TCA) [3,19].

Diverse group of substrates like glucose, sucrose, succinate and volatile fatty acids (VFAs), as carbon sources, could be catalyzed by photosynthetic non-sulfur (PNS) bacteria to produce hydrogen gas. Some of common PNS bacteria, which are used in photo-fermentative H_2_ production processes, are namely: *Rhodobacter sphaeroides* O.U001, *Rhodobacter capsulatus*, *R. sphaeroides-RV*, *Rhodobacter sulfidophilus*, *Rhodopseudomonas palustris* and *Rhodospirillum rubrum* [12].

Application of a non-sulfur purple photosynthetic bacterium is another promising fermentative process, in which complete conversion of substrate to hydrogen and CO_2_ could be achieved. This bacterium is capable of producing ATP and high energy electrons under anaerobic condition. Nitrogenase gets involved in the next step to produce hydrogen by proton reduction through using ATP and reduced ferredoxin. This bacterium is not powerful enough to split water like cyanobacteria and/or green algae in photolysis process. Therefore, organic substrates (organic acids) are necessary as electron donors [20]. Besides the important aspects of this process, there are some disadvantages like high energy demand of nitrogenase, low light conversion efficiencies, and expensive hydrogen separation processes, which need additional effort of researchers to find solutions for these problems [21].

#### 2.1.2. Dark Fermentation

Dark fermentation is one of the powerful processes of biohydrogen production through biomass conversion due to its higher rate of hydrogen production and easier operation in comparison to photo-fermentation. In dark fermentation, a catabolic process occurs using anaerobic bacteria without any need of direct solar input [22,23]. Substrates like carbohydrates and proteins are converted into carboxylic acids, hydrogen gas, carbon dioxide and organic solvents by bacteria grown in the dark [20,24]. Production of acetic, butyric and other organic acids is the main problem of dark fermentation, because it is responsible for lowering the hydrogen production by shifting the metabolic pathway [25].

Since the presence of oxygen is the main inhibitor of this process, thus the anaerobic condition must be provided. *Escherichia coli* [26], *Clostridium* and *Enterobacter* species are common anaerobic bacteria that are used for hydrogen production from organic substrates. Since maintaining the strict anaerobic condition is difficult, facultative anaerobic bacteria, such as *Escherichia coli* and *Enterobacter* species, are preferred. These anaerobes are less sensitive to dissolved oxygen, and the activity of the enzyme involved in hydrogen production is not damaged by the presence of trace of oxygen in the medium [2]. Acetate, butyrate, carbon dioxide and organic solvents are other byproducts of dark fermentation process. Different organic substrates and wastewaters can be used as electron donors in biohydrogen production with lower operational cost compared to other processes. Complex organic polymers can be also hydrolyzed and converted into a mixture of simple organic acids, alcohols and hydrogen by hydrogen producing bacteria without light [17].

Product distribution in dark fermentation process can be varied based on effective parameters such as the oxidation state of the substrate, microbial distributions and environmental conditions such as pH, temperature and hydrogen partial pressure [10,21]. According to operating temperature conditions for microbial cultures, hydrogen production studies are conducted in four temperature regimes: ambient (15–30 °C), mesophilic (30–39 °C) and thermophilic (50–64 °C) and hyper-thermophilic (>65 °C). For example, according to the results reported in the literature both strains, *Rps. palustris P4* and *Citrobacter sp. Y1* can produce hydrogen from the sugars under a wide range of pH (5–9) and temperature (25–40 °C), giving the maximum hydrogen yields of 2.8 mmol-H_2_/mmol-glucose [27] and 2.5 mol-H_2_/mol glucose [28], respectively. Mandal et al. [29] demonstrated that biohydrogen production is under influence of H_2_ partial pressure. When the partial pressure of H_2_ was decreased by lowering the total pressure in the headspace of the reactor in a batch fermentation process from 760 to 380 mm Hg containing *Enterobacter cloacae*, the molar yield of H_2_ increased from 1.9 to 3.9 mol H_2_/mol glucose. Consequently, the optimum operational conditions to reach maximum hydrogen production is completely dependent on the microbial culture used for the fermentation process.

Pyruvate is the main intermediate product formed during the catabolism of carbohydrate rich substrates. Hydrogen can be produced through catalyzing pyruvate by one of two enzyme systems mentioned below [30]:

1. Pyruvate: formate lyase (PFL) Pyruvate + CoA → acetyl-CoA + formate
2. Pyruvate: ferredoxin oxido reductase (PFOR) Pyruvate + CoA + 2Fd(ox) → acetyl-CoA + CO_2_ + 2Fd(red).
3. $\mathrm{acetyl}-\mathrm{CoA}+\mathrm{formate}\overset{FHL}{\to }{\mathrm{CO}}_{2}+H_{2}$

The maximum biohydrogen production yield for strict anaerobic bacteria is reported to be 4 moles of hydrogen per mole of glucose, while this yield is about 2 moles of hydrogen per mole of glucose for facultative anaerobes [3]. 

### 2.2. Biophotolysis

Biophotolysis is a process attributed to photoautotrophic organisms such as microalgae and cyanobacteria, which are capable of generating hydrogen and carbon dioxide under anaerobic condition [31]. Biohydrogen is a product of the water splitting by using these organisms under light as energy source and carbon dioxide as carbon source [15]. Some advantages of biophotolysis are the simplicity of the process to produce hydrogen without needing additional substrates as nutrient and the direct production of hydrogen using abundant source of water as electron donor, sunlight and carbon dioxide as the main sources of energy and carbon for the growth of microalgae and cyanobacteria [32]. Biophotolysis is classified into two groups: direct biophotolysis and indirect biophotolysis. 

#### 2.2.1. Direct Biophotolysis

In direct biophotolysis water is converted into hydrogen and chemical energy under light irradiation as reported in Equation (3) [33]. This process is similar to processes taking place in plants and algal photosynthesis [34].

(3) $$2H_{2}O\overset{hv}{\to }2H_{2}+O_{2}$$

In Equation (3), *hv* represents the energy from a photon in light (h is the Planck constant and v is the frequency of the light). Ferredoxin, photosystem I (PSI) and II (PSII) are all involved in conversion of light into chemical energy as H_2_ molecule. The absorbed light energy is used to transport electrons linearly to ferredoxin. The mechanism of electron transfer via the hydrogenase enzyme is demonstrated by the following reactions, Equations (4) and (5) [35]:

2H_2_O →4H^+^ + 4e^−^ + O_2_ (4)

4H^+^ + 4e^−^ →2H_2_ (5)

Hydrogenase converts H^+^ into H_2_ by accepting electrons directly from reduced ferredoxin to generate H_2_. This process is referred to the algae photosynthesis system through which the biohydrogen is produced by one-stage direct biophotolysis using water and solar energy [32]. Oxygen sensitivity of hydrogenases enzyme used in hydrogen production is the main constraint of this process that inhibits the activity of the enzyme, with consequent lower hydrogen yields reached. However, a two-stage direct biophotolysis is proposed to overcome this limitation. In this method, oxygen is eliminated through the respiration using exogenous or endogenous substrates such as sulfur deprivation method. Considering the oxygen sensitivity of involved enzymes, the hydrogen production yield in the order of 0.07 mmol/L h was reported in the literature [3].

#### 2.2.2. Indirect Biophotolysis

Hydrogen production via indirect biophotolysis process consists of two stages, coupled in series. The first stage is the biomass production through photosynthesis of carbohydrate as substrate, and the second stage is the fermentation of the biomass rich-carbohydrate for hydrogen production as shown below [36]:

6H_2_O + 6CO_2_ + light energy→C_6_H_12_O_6_ + 6O_2_ (6)

C_6_H_12_O_6_ + 2H_2_O →4H_2_ + 2CH_3_COOH + 2CO_2_ (7)

Following these steps leads to temporally and/or spatially separation of the oxygen and hydrogen evolutions, which prevent enzyme deactivation and explosive property of the gas mixture [37]. A simple separation method is required for hydrogen purification since CO_2_ can be easily removed from the produced H_2_/CO_2_ mixture [33].

*Gloebacter sp*., *Synechocystis sp*., and *Synechococcus sp*. are some species of cyanobacteria organism that can be used in indirect biophotolysis process [5]. Anabaena species and strains are commonly used due to their high hydrogen production rates. Furthermore, mutant strains of *A. variabilis* are also capable of participating in indirect biophotolysis process with hydrogen production rate in the order of 0.355 mmol/h per liter [3].

## 3. General Features of MBR Systems

An MBR is described as a biological reactor forecasting the inclusion of a membrane unit inside. Therefore, it is considered to be a hybrid solution, combining a biological system with the membrane filtration, meanwhile the reactor design and process configuration affect the overall chemistry of the hydrogen production process. In this respect, several new configurations of experimental setups have been developed to optimize the hydrogen production rates and yields. 

### 3.1. Bioreactor Configurations

The main classification of MBRs is according to biological treatment performance and the microbial structure, the latter classified as aerobic MBRs (AeMBRs) and AnMBRs. In AnMBR systems, the electron acceptor can be CO_2_ or part of the organic matter itself, obtained as a product of this reduction, or the carbon in its most reduced state [38]. AnMBRs are similar to AeMBRs, differing for the absence of any aeration system inside the first typology and for the low biomass production. AnMBRs were widely used in wastewater treatment even at full-scale plants. The anaerobic system is slower than the aerobic one in terms of chemical oxygen demand (COD) reduction, but they do not need any energy supply for aeration [39,40]. In brief, the advantages of AnMBRs may be summarized as the lower energy demand due to the absence of aeration, the possibility of operating in bubble coarse mode and recycling of the headspace gas using spargers, reduction of the cake layer development by recycling gas flow, improvement of the liquid-to-gas mass transfer rate by continuous gas flushing, and reduced biomass production and its associated disposal costs [19,41,42]. The main drawback of the anaerobic treatment is the lower quality of effluent generated, especially when operating with low-strength wastewaters [43,44]. Overall, AnMBRs are desirable systems for dark fermentative hydrogen production since the activity of hydrogenase enzymes is sensitive to increasing H_2_ concentrations in the aqueous phase [41,42,44,45,46].

According to the membrane location in the bioreactor system, there are different classifications of MBRs. Sidestream and submerged configurations are two basic types of MBRs. In the sidestream system, the membrane module is placed in a pressurized circulation loop located outside the bioreactor. The mixed liquor in the reactor is pumped through the circulation loop containing the membrane unit and the permeate is discharged and retentate is recycled to the bioreactor. In submerged MBR, membrane unit is directly submerged inside the bioreactor tank and the driving force across the membrane is achieved by applying a lower pressure than on the permeate side of the membrane [40,47]. The sidestream configuration is generally applied to the treatment of high-strength wastewaters (e.g., industrial wastewater), while the immersed configuration is usually applied to the treatment of low-strength wastewaters (e.g., sewage). The basic advantage of sidestream configuration is the possibility of physical cleaning of the membrane surface due to the present cross-flow. Nevertheless, high energy is needed to reach the desired cross-flow velocities in the sidestream configuration. Low energy requirement is the main advantage of submerged configuration of MBR since the permeate could be driven by vacuum or gravity force. Besides, in submerged MBRs, the control of membrane fouling is possible by biogas-assisted membrane scouring.

In order to improve the biohydrogen production, different bioreactor configurations are proposed and studied. Robust systems with reliable performance, high stability during long time operations (months) and reactors with high resistance to fluctuations of operational parameters are preferred for hydrogen production. Both batch and continuous modality could be used for biohydrogen fermentations, while for industrially feasible processes, continuous or semi-continuous mode is applicable. Continuous stirred tank reactors (CSTRs) were widely used for hydrogen production due to their simple construction, ease of operation and high homogenous mixing ability. Hydraulic retention time (HRT) is the most effective parameter in these reactors since it controls the microbial growth rate and biohydrogen production rate as well. Different bioreactor setups used for hydrogen production are summarized in Table 1. As reported in Table 1, since the reactor configurations and operating conditions may differ a lot, it is difficult to evaluate which of them may represent a more favorable system for biohydrogen production. However, a comprehensive study would be necessary for each kind of reactor reported in Table 1 operated at different conditions in order to make reliable and reasonable deductions.

### 3.2. Membrane Materials

Various materials and configurations of membrane modules are used in full-scale MBRs, which are commonly classified on the basis of membrane pore size. Flat sheet (FS), hollow fiber (HF), and multitubular (MT) are various membrane modules that are commonly used [39]. Typical pressure-driven filtration types that could be used in MBRs include dynamic membrane filtration, microfiltration (MF), ultrafiltration (UF), and nanofiltration (NF). The range of pore sizes is relatively small for these membranes, which is 0.1–1 µm for the MF, 0.01–0.1 µm for UF, and 1–10 nm for NF. The applied transmembrane pressure (TMP), as the important parameter in membrane systems, ranges from 10 to 80 kPa for HF or FS membranes in submerged MBRs, while it is from 20 to 500 kPa for MT membranes in external MBRs [60,61].

On the basis of membrane material, polymeric and ceramic membranes are generally used in membrane production systems. Some of the most used polymeric membranes are based on polyvinylidene difluoride (PVDF), polyethersulfone (PES), polyethylene (PE), polypropylene (PP), and polysulfone (PS) [62]. Due to hydrophobic nature of the aforementioned polymers they should be modified to contain hydrophilic groups before used in MBRs. Ceramic membranes, as inorganic membrane materials, could be also used in MBR systems. They have good thermal and chemical stability, and high resistance to corrosion, abrasion and fouling. These properties increase the efficiency of backwashing and improve their durability over polymeric ones. However, ceramic membranes are more expensive than polymeric and this fact leads to limited utilization of ceramic membranes in MBRs. Today, FS ceramic membrane modules are receiving significant attention [39]. UF and MF ceramic membranes are the most used in thermophilic membrane bioreactors (ThMBR) due to their high thermal stability, although polymeric membranes were also used.

As shown in Table 2, a large range of membrane fluxes, from 7 to 72 L m^−2^ h^−1^ (LMH) and from 2.2 to 80 LMH are reached for thermophilic aerobic MBRs (ThAeMBRs) and thermophilic anaerobic MBRs (ThAnMBR), respectively. Comparable fluxes are obtained for both the thermal reactors and the large range could be attributed to different parameters such as wastewater characteristics, type of membrane, and operating conditions, which may affect considerably the membrane flux [63].

### 3.3. Potentials and Limitations of AnMBR Technology

Since AnMBR is a widespread technology used for wastewater treatment and biohydrogen production, a comprehensive study about the benefits and constraints is of particular interest in order to identify how to operate an AnMBR-based process. Some advantages of AnMBR technology are summarized as reported below [16]:

- Ability of retaining anaerobic microbes due to uncoupling of HRT and solid retention time (SRT).
- Providing cost-effective and environmental friendly ambient-temperature anaerobic digestion (AD) by increasing SRT [74].
- Low disposal of biosolids due to low growth yield of anaerobic biomass.
- Improving the stabilization of biosolids by increasing SRT.
- Employing micron pore-sized filtration as appropriate membrane cut-off, which leads to production of high-quality permeate.
- Ability of conversion of biodegradable compounds into gaseous energy carriers like methane and biohydrogen at ambient temperature [30].
- Reducing greenhouse gas emissions by saving energy consumption.

Nevertheless, AnMBR technology presents some issues that are responsible for the lower process efficiency, limiting its applicability in various operations. Some of these constraints are summarized below:

- Increase of methane concentration in the effluent by decreasing temperature, which needs consequently to be captured before its stripping to the atmosphere [75].
- Membrane fouling and cleaning frequency [76].
- Necessity of applying a post-treatment step for nutrient removal/recovery [77].

**Figure 1:**
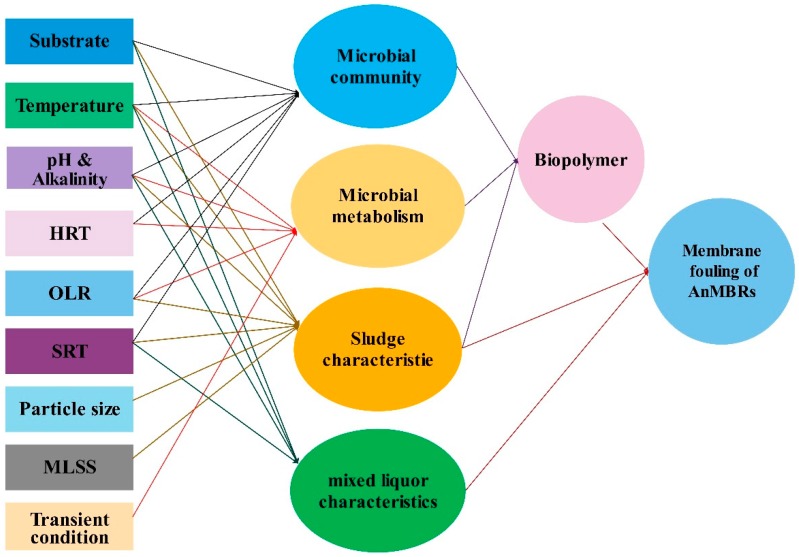
The operational factors affecting membrane fouling in AnMBRs.

Although many progresses were reported in AnMBRs application for wastewater treatment and hydrogen production, membrane fouling is still the main barrier of application of AnMBRs at large-scale. Membrane fouling is generally associated with the deposition of solids like sludge flocs and the adsorption of dissolved organic matter on the membrane surface [78]. Membrane fouling could lead to reduction of the membrane’s permeability. Since cleaning frequency is a function of the flux rate, membrane fouling increases the operating frequency of membrane cleaning, reducing the membrane′s lifetime. This negative phenomenon is influenced by various factors such as microorganism community, substrate characteristics, concentration of extracellular polymeric substances (EPS), membrane type, and operating conditions [38]. Figure 1 illustrates schematically the various parameters affecting the membrane fouling of AnMBRs. By varying the microbial metabolism conditions (lowering the HRT), increasing SRT and higher feed strength could result in higher biopolymers production like soluble microbial products (SMP), which prevents membrane fouling. 

## 4. Biohydrogen Production in Anaerobic Membrane Bioreactors

Reactor design and process play key role in achieving high biohydrogen formation capacity. Therefore, it is important to choose the best bioreactor system to provide the appropriate microenvironment, hydrodynamic behavior, prevailing microbial population and their contact with the substrate [79]. CSTR is the most common reactor type used in the literature. Popularity of CSTR is due to its well-mixed medium condition and perfect mass transfer, which induce the proper contact between the microorganisms and the substrate. Nevertheless, washout phenomenon at shorter HRTs is the main issue of these reactors that causes lower hydrogen generation rates. On the other hand, higher biohydrogen production rates could be achieved in AnMBRs even at higher levels of HRTs compared to CSTRs. This advantage is due to the membrane application in AnMBRs, which leads to retention of biomass in the reactor medium and less sludge production [31,62,80].

### 4.1. Factors Affecting Biohydrogen Production in AnMBRs

Biohydrogen production rates and yields in AnMBRs are influenced by various parameters which are summarized in this sub-section.

#### 4.1.1. Substrate Concentration and Nutrients Loading

Efficient microbial growth is dependent on the availability of necessary nutrients like carbon source as substrate, nitrogen, phosphate and inorganic trace minerals. Various substrates could be used as source of carbon and energy and bioconverted into molecular hydrogen gas by the catalytic activity of the hydrogen producing enzyme [8]. Since high- and low-strength wastewaters could be used as substrate in AnMBRs, the presence of some supplemental elements is essential to reach optimal growth and biohdrogen production as well. Different concentration of nutrients may influence on the H_2_ production efficiency and have inhibitory effects at high concentrations. 

Organic nitrogen and iron element play key role in hydrogen production process and improves AnMBR efficiency. Iron helps to mediate between hydrogenase and nicotinamide adenine dinucleotide (NADH)-ferredoxin reductase [81].

Substrate/organic loading rate (OLR) is a key factor in design of hydrogen formation biosystems in AnMBRs. Various researches were conducted to obtain the optimum OLR in terms of maximization of hydrogen generation efficiency. However, a definitive answer has not been provided since the systems are carried out in different operating conditions and fluctuating circumstances. As reported in the literature, the gradual increase of OLR (from 4 to 22 g COD/L-d) has a positive effect on hydrogen production efficiency of the AnMBR, but high OLRs (30 g COD/L-d) lead to a significant decrease (20%) in the gas generation performance [46,82].

The substrate concentration is the other factor affecting the hydrogen production yield. It is reported that it is increased with an increase of glucose concentration from 10 to 35 g/Lat HRT of 12 h. Nevertheless, reverse effect is seen for sucrose as substrate. The hydrogen production yield is decreased from 1.7 mol H_2_ mol^−1^ hexose to 0.8 mol H_2_ mol/L hexose by increasing hexose concentration from 10 to 50 g/L, respectively. This result could be attributed to interactions present between the substrate concentration and other effective parameters such as HRT, SRT, and different composition of microbial cultures [17,83].

#### 4.1.2. Hydraulic Retention Time (HRT) and Solid Retention Time (SRT)

Generally, HRT and SRT affect the substrate degradation efficiency, microbial growth and production rate of a biosystem as well. However, evaluating the optimum HRT and SRT seems essential for all the bioreactors. The effect of different SRT and HRT levels on H_2_ generation performance of various AnMBRs are summarized in Table 3. According to the literature, the HRT and SRT values have an adverse effect on biohydrogen yield and volumetric productivity. In order to prevent the probable biomass washout, HRT must be adjusted at levels higher than maximum microbial growth rate [21]. Retention of biomass is possible via solid–liquid separation using appropriate membranes in AnMBRs. This will lead to decoupling HRT and SRT in this biosystem, with consequent several benefits compared to CSTRs [84].

According to previous studies, in AnMBRs, a decrease of HRT may cause inhibition of methanogenesis and thus may improve the hydrogen production rate [56,85]. Moreover, reducing HRT may inhibit the propionate generation by reducing microbial diversity and may result in an increase in the biohydrogen yield [86,87]. According to these results, the relatively short hydraulic retention times that have been previously studied amply demonstrate that maintaining shorter HRT and longer SRT is the preferred solution for biohydrogen producing microorganisms and might improve the bio H_2_ generation efficiency [88]. Anaerobic hydrogen production via membrane bioreactors are appropriate cell-retention devices designed to ensure a sufficiently long SRT which increases the hydrogen formation capacity [46].

#### 4.1.3. Temperature and pH

Since various microbial communities and enzymes are involved in biohydrogen production process, their activity is under the influence of temperature and pH as key operating parameters. Although ambient temperature is preferred for a wide range of hydrogen producing microorganisms, higher temperatures could be better for mesophilic microbial communities, improving the H_2_ production yield [8]. In addition, hydrogen gas solubility in broth is reduced at higher temperatures. This fact enhances hydrolysis and restricts activity of propionic acid and methane, producing enzymes. These phenomena result in boosting of H_2_ production efficiency. The temperature range from 15 to 34 °C was tested for a mixed culture by Chang and Lin [49]. Maximum hydrogen yield and specific hydrogen production rate of 1.42 mol H_2_ mol^−1^ glucose and 359 mmol L^−1^ d^−1^ was achieved at higher range of temperature (30–34 °C), confirming the above mentioned discussion. The effect of temperature was also verified by Sivagurunathan et al. [95], who demonstrated the feasibility of an increase of 62% in biohydrogen production yield by raising the temperature from 37 to 45 °C.

Similarly, pH is another important variable strongly affecting the metabolic pathway, cell morphology and structure, microbial population shift and yield of biohydrogen. The undesired activity of propionogenesis and methanogenesis could be limited by changing pH. These enzymes consume the produced biohydrogen and reduces the biohydrogen production performance [95,96]. Restriction of methanogens is possible at cultivation pHs below 4.5. The optimum pH value for biohydrogen production process seems to be between 5.2 and 6.0, using pure or mixed microbial cultures, guaranteeing maximum H_2_ yield and production rate [8,56]. Mohan [86] found pH values between 5.5 and 7.5 as the optimum pH range for hydrogen production, showing lower production rate outside the aforementioned optimum range. However, in the literature various studies revealed that the optimum pH range may vary depending on the physiological characteristics of substrate and composition of microbial population [11,97]. 

#### 4.1.4. Hydrogen Partial Pressure

During the fermentative process in AnMBRs, the dissolved hydrogen concentration increases by increasing of hydrogen partial pressure. This phenomenon is undesirable since it influences the microbial pathways and metabolic flux and leads to formation of lactate and other solvents (such as ethanol, acetone butanol). Therefore, hydrogen production yield is suppressed in this way and removing excess hydrogen seems mandatory to maintain hydrogen production in the system [17,98]. These results are achievable by sparging nitrogen gas in the system. As reported by Mizuno et al. [98], the negative effect of hydrogen partial pressure is overcome and increased up to 65% when the nitrogen sparging is applied in the reactor. In other studies, an increase of 68% (0.85 to 1.43 mol H_2_ mol^−1^ hexose) was achieved by sparging N_2_ gas in the system [17]. The only constraint of this technique is constituted of the reduced purity of biohydrogen due to dilution impact of N_2_ gas [4].

#### 4.1.5. Microbial Culture and Metabolism

Different pure or mixed cultures are used for biohydrogen production. Generally, batch mode process is used using pure cultures in aseptic condition. Metabolic engineering is done on *E. coli* strain as a common used hydrogen producing bacterium to achieve the maximum biohydrgen production yields. Various sugars can be consumed by *E. coli* as substrate to produce hydrogen. Using additives and mutated strains of *E. coli*, it is possible to reach maximum production yield of 2 H_2_/glucose for this organism. This bacterium is used for engineering purpose since its genome can be easily manipulated. Enhancement of hydrogen production by existing pathways is possible by increasing the flux through gene knockouts of competing pathways or increasing homologous expression of enzymes involved in the hydrogen-generating pathways. Various phenomena in metabolic pathway of *E. coli* result in increase of H_2_ production yield, which are briefly described as follows: (a) inactivation of a pathway that drains the pyruvate pool; (b) inactivation of lactate dehydrogenase or fumarate reductase; (c) activation of formate–hydrogen lyase to degrade formate to H_2_ and CO_2_; (d) expression of enzymes, such as formate dehydrogenase H and hydrogenase [21].

*Clostridium* and *Enterobacter* are extensively adopted as inoculums, using glucose as substrate for fermentative hydrogen production. This process benefits high selectivity and yield since manipulation of microbial metabolism is possible by varying and optimizing growth and operational conditions [99]. Despite the benefits of pure culture, using mixed culture microbial population is preferred in most of the previous studies since they are applicable in non-sterile condition, which reduces the overall operation cost. Applicable mixed cultures can be obtained from anaerobic sludge, municipal sewage sludge compost and soil [8,100,101]. Robust mixed cultures are more flexible against varying operational conditions and can support diverse metabolic activities. Non-hydrogen producing bacteria could be involved in mixed cultures, thus appropriate operating conditions or pretreatment steps are required to restrict the competing bacteria and increase the predominance of hydrogen producing bacteria [17,21,96,102].

## 5. Membrane Fouling and Fouling Mechanisms

Fouling is a long-standing challenge and common drawback in AnMBRs, needing consideration for its effect on the membranes. Fouling is usually related to formation of a cake layer on the membrane surface, or deposition/adsorption of dissolved particles within membrane matrix, due to the physicochemical interaction among the mixed liquor and the membrane in AnMBR. These phenomena lead to blocking of membrane pores, deterioration of membrane permeability and flux decline [38].

Several parameters are effective on membrane fouling during hydrogen production in AnMBRs, which can be subdivided in four main categories: membrane characteristics (membrane configuration, material, hydrophobicity, porosity, pore size), biomass and mixed liquor properties (MLSS, EPS, SMP, floc structure and size, dissolved matter), operating condition (MBR configuration, cross-flow velocity, aeration, HRT, SRT, TMP) and the properties of sewage [103]. Temperature is one of the challenging parameters for AnMBRs operating at ambient temperature. Fouling is more probable for these reactors operating in cold regions or winter season due to increase of liquid viscosity. Therefore, a high viscosity induces a higher drag force towards the membrane, and mixing will get harder as well, globally requiring more energy for preventing fouling [104,105,106]. Furthermore, higher rates of membrane fouling is observable in ThMBRs compared to the mesophilic ones [107,108,109]. This could be attributed to formation of more EPS in ThMBRs due to the deflocculation nature of thermophilic sludge [110]. The MLSS concentration is another key parameter controlled by SRT. Long SRTs and high suspended solids concentration in the influent result in high MLSS concentration, which intensifies membrane fouling [111,112]. As studied in the literature, pH also affects membrane fouling rate. A significant increase in UF membrane fouling rate was reported by Sweity et al. [112] by increasing the pH from 6.3 to 8.3. This is due to the low adherence and fouling propensity of EPS at low pH values [113].

**Figure 2:**
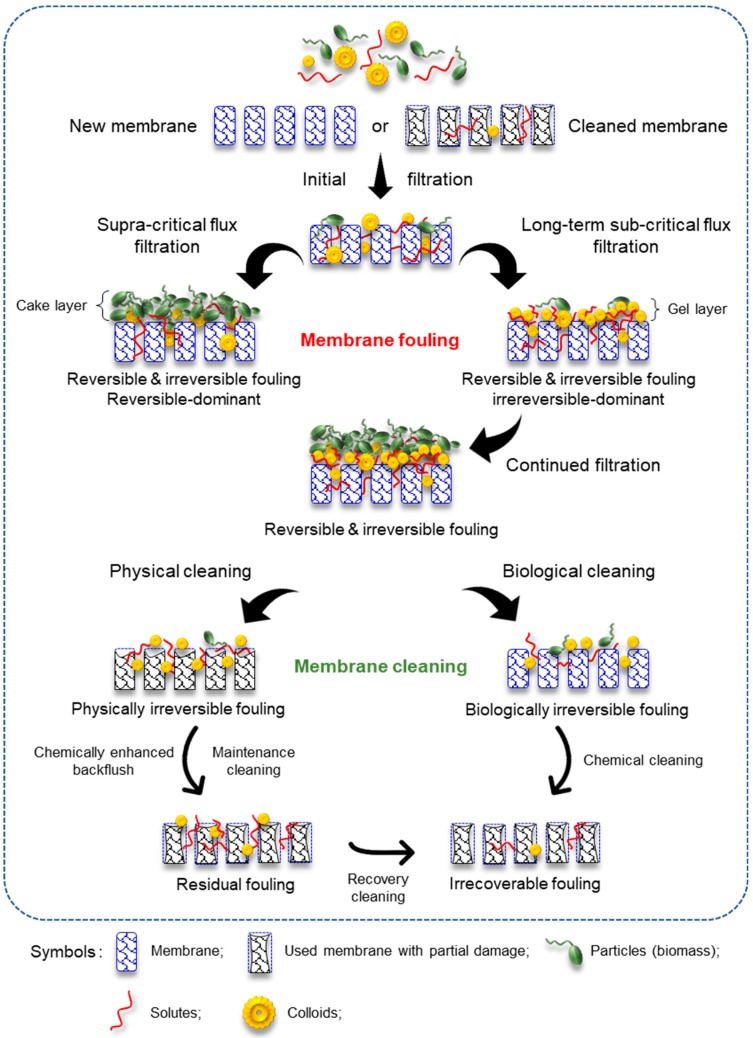
Overview of membrane fouling types, mechanisms and the required cleaning techniques.

Fouling could be determined by monitoring TMP and flux changes for membranes operating at constant flux or pressure mode, respectively. Increasing TMP and decreasing flux is indicative of high resistance of and membrane fouling in the system [114,115,116]. Various compounds contribute to this effect such as particulates, organics, colloids, microbes and microbial byproducts, biofilm including EPS and dissolved inorganic compounds [117]. All types of membrane fouling, mechanisms and the required cleaning techniques [118,119,120] are summarized in Figure 2 and are discussed in detail in the following sections.

### 5.1. Biofouling, Organic/Inorganic Fouling

The main mechanisms responsible for membrane fouling are pore narrowing, pore blocking, cake formation, concentration polarization, organic adsorption, inorganic precipitation and biofouling. Fouling classification is based on the biological and chemical characteristics of membrane foulants. The membrane cleaning methods are chosen according to the nature of membrane foulants, which significantly affect the membrane cleaning efficiency [76]. Biofouling, organic fouling, and inorganic fouling as common membrane fouling formation mechanisms in AnMBRs are discussed in the following subsections.

#### 5.1.1. Biofouling

Biofouling is the most complicated fouling mechanism since it is caused by undesirable deposition, growth and metabolism of bacterial cell/cell cluster or flocs on membrane surface and/or inside membrane pores. Hence, membrane surface is colonized by microorganisms and when this happens, the result is called biofilm or biocake. Biosolids are dominant foulants for sludge cake and are responsible for a significant concern in membrane filtration process [121,122]. Since membrane pore sizes in MF and UF systems are smaller than most of the microbial flocs in AnMBRs, pore plugging is a major problem in these systems. SMPs and EPSs produced by microbial culture, which are commonly classified as organic foulants, significantly affect formation of biocake and hence biofouling on membrane surface [123,124].

As reported in some publications, some microbial communities used in MBRs are pioneers of surface colonization and play an important role in membrane fouling. For example, the *Betaproteobacteria* are a specific phylogenetic group of bacteria that develop mature biofilms, leading to severe irremovable membrane fouling [125]. Therefore, it seems crucial to have a comprehensive study on deposition behavior of bioflocs/cells and their attachment mechanism to develop proper biofouling control strategies in AnMBRs [126].

#### 5.1.2. Organic Fouling

Organic fouling is caused by deposition of small size of biopolymers such as proteins, polysaccharides, humic acids and other organic substances (either soluble or colloidal) on the membrane surface [127]. These depositions might be originated from feed water or microbial secretion. The adsorption of organic matters produced by microorganisms e.g., SMP and EPS are also considered as organic fouling in AnMBRs. Chelating polymers (organic-inorganic complexes) can be produced via interaction between metal cations and functional groups of biopolymers like calcium alginate, which can cause severe fouling in MBRs [76]. The gel fouling layer formed by organic foulants is composed of different layers. Based on fractionation results, the upper layer composition was found to be similar to sludge flocs, while high concentration of polysaccharides, SMP and bacterial colonization were observed in intermediate layers. SMP and high concentration of bound proteins are the predominant compounds found in lower layers as the irremovable fouling fraction [128]. Generally, SMP or EPS could be considered as the origin of organic fouling and their deposition strongly depends on their affinity with different membranes [126].

#### 5.1.3. Inorganic Fouling

In general, inorganic fouling is represented by a chemical deposition of inorganic elements (Ca, Mg, Al, Si, etc.) and/or biological precipitation of inorganic-organic complexes. The metal ions and anions such as CO_3_^2−^, SO_4_^2−^, PO_4_^3−^ and OH^−^ might react and cause chemical precipitation. The precipitation occurs when the concentration of chemical species exceeds the saturation concentrations due to concentration polarization on the membrane surfaces [76]. The formed precipitants might attach the membrane surface and block membrane pores, producing the inorganic fouling. Biofouling and organic fouling are more relevant in AnMBRs than inorganic fouling, but the former could happen also concurrently in membrane MBRs. As reported in some publications, high alkalinity of activated sludge (pH = 8–9) causes precipitation of CaCO_3_ and, hence, severe inorganic fouling in MBRs [129]. Intense CaCO_3_ fouling was observed by Ognier et al. [129] in a MBR with a ceramic UF membrane module. In the fouling layer characterization, by Kang et al. [130], *Struvite* (magnesium ammonium phosphate, MgNH_4_PO_4_·H_2_O) as an inorganic foulant, was observed in the thick cake layer [130,131]. Metal carbonates (Ca, Mg, and Fe ions) might be also formed using CO_2_ produced during anaerobic fermentation. These salts affect pH of the liquid medium and lead to membrane scaling [132]. Metal ions could also be caught by the biocake layer via complexing and charge neutralization by passing across the membrane. However, formation of fouling layers are enhanced in this way and metal ions can bridge the deposited cells and biopolymers and form a dense cake layer as well. Consequently, a synergistic interaction among biofouling, organic fouling and inorganic fouling is worthy of note.

### 5.2. Reversible, Irreversible, Residual, and Irrecoverable Fouling

The other classification of membrane fouling is based on the attachment strength of particles to the membranes or the method used to recover the initial permeability of the membranes. Four types of fouling are defined accordingly, namely reversible, irreversible, residual, and irrecoverable fouling, as in Table 4 and discussed below [110]:

#### 5.2.1. Reversible Fouling

Weak attachment of foulants to membrane surface results in reversible or temporary fouling. This fouling type is removable by physical cleaning strategies such as relaxation, backwashing or air scouring for low-pressure systems. Biosolids present in the cake layer are considered as the main source of reversible fouling. The degree of fouling reversibility strongly depends on the frequency, duration, and strength of the cleaning method. However, enhanced physical strategies could be applied to remove the long-term developed reversible fouling [76,126].

#### 5.2.2. Irreversible Fouling

During continuous filtration operation, various solutes could form a strong matrix of foulants like gel layer, responsible for an irreversible fouling and requiring chemical reagents to be mitigated. Pore narrowing or pore plugging are also classified as irreversible fouling type. Organic matters, especially polysaccharide-like organic matter, play an important role in the development of the irreversible fouling. Physical cleaning methods are not applicable in removing irreversible fouling and chemical or biological cleaning methods are proposed in the literature to overcome irreversible fouling [76,131]. Although internal residual fouling caused by pore narrowing or blocking and strongly attached layers (gel or dense cake layers) can be removed by chemical cleaning, unfortunately the demand for frequent chemical cleaning can deplete the membrane lifetime.

#### 5.2.3. Residual Fouling

Residual fouling concept was proposed by Kraume et al. [133] and Judd [134]. The residual or permanent fouling is formed on the fresh membrane surface through adsorption by strong electrostatic/hydrophobic attractive forces and hydrogen bonds. Accumulation of residual foulants after chemical cleaning is the main source of residual fouling. The residual resistance remaining after cleaning increases with the number of fouling/cleaning cycles until a maximum is reached. This phenomenon results in a more tightly structured and oriented monolayer attachment of macromolecules of proteins and polysaccharides, which could be able to withstand chemical attack. Therefore, chemical cleaning could not eliminate the residual fouling formed on the virgin membrane surface. Furthermore, it cannot be removed by chemically enhanced backflush or maintenance cleaning since the caustic swelling-to-breaking mechanism is simply not able to decompose the residual fouling. On the contrary, the recovery cleaning and oxidation could be effective for the removal of residual fouling [76,135]. As reported by Gan et al. [135], combined caustic cleaning and peroxide oxidation is used for removal of residual fouling. Since the divalent and hydrogen bonds are susceptible to oxidative attack, bond cleavage occurred during oxidative attack, resulting in breaking-up of foulants formed on the fresh membrane surface.

#### 5.2.4. Irrecoverable Fouling

The term irrecoverable fouling refers to fouling that cannot be removed neither by physical nor by chemical methods and occurs after a long operational period. The original virgin membrane permeability could never be recovered after fouling during normal operation. The remaining resistance is defined as irrecoverable or permanent fouling, which cannot be removed by typical cleaning methods. Since the rates of irrecoverable fouling are between 10^−4^ to 10^−3^ mbar/min thus, it builds up over several years and take away from the overall membrane life [136,137].

### 5.3. Strategies for Fouling Removal

Membrane fouling is one of the biggest challenges in the widespread application of MBRs where interactions between constituents of activated sludge and membranes causes fouling. While comprehensive understanding of fouling and its mechanisms may be useful in many aspects, and the control and removal of fouling is essential as well. Removal methods generally consists of six main strategies: (1) using appropriate pretreatment to the feed water, (2) decreasing the flux, (3) increasing the aeration, (4) employing appropriate physical or chemical cleaning protocols, (5) chemically or biochemically modifying the mixed liquor and (6) membrane surface modification [134].

#### 5.3.1. Physical Cleaning

Simple cleaning techniques such as relaxation and membrane backwashing/backflushing (where permeate is pumped in the reverse direction through the membrane) are considered as physical cleaning methods to limit fouling. The relaxation and backwashing conditions also considerably affected the fouling rate. The reversible fouling caused by pore blocking and loosely attached cake could be successfully removed by applying backwashing. Backwashing is effective on clogging near the membrane surface and can lose or remove the formed layer. However, vigorous backwashing is not an appropriate removal technique for flat plate submerged membranes and may damage the membrane. Backwashing duration, interval, and strength are key factors affecting the efficiency of this technique, which are significantly correlated with the amount of solids and soluble fractions deposited on the membrane surface [115,138,139]. Increasing the backwashing duration and frequency, the backwashing efficiency would increase, but it seems essential to optimize backwashing based on energy and permeate consumptions. Regarding the TMP value, less frequent but longer backwashing durations (e.g., 600 s filtration/45 s backwashing) are preferred [115,140]. Air can also be used as the backflushing medium, but continuous application of air backwashing may cause membrane breakage and rewetting. Flux enhancement of 400% could be obtained by applying backwashing (15 min filtration/15 min air backwashing) in MBRs. For HF systems, backflushing, if employed, is usually applied at fluxes of 1–3 times the operating flux [141].

Membrane relaxation encourages diffusive back transport of foulants away from the membrane surface under a concentration gradient, which is further enhanced by the shear created by air scouring. Relaxation is commonly used in submerged MBR systems for either hollow fiber or flat-sheet membranes and improves membrane productivity. It is typically applied for 1–2 min every 8–15 min of operation. Membrane relaxation allows filtration to be maintained for longer periods before the need for chemical cleaning arises. However, a too long and a highly frequent relaxation would cause critical fouling due to the relatively high instantaneous flux. Although, relaxation is considered not to be economically feasible for large-scale MBRs, this method is almost ubiquitous in modern full-scale submerged MBRs/AnMBRs due to results of cost and productivity analyses. Further attempts are being made to combine relaxation with backflushing for optimum results in fouling removal [115].

#### 5.3.2. Chemical Cleaning

By accumulation of irreversible fouling on membrane surface during operational process, application of physical cleaning methods seems not to be adequate and the utilization of different chemical cleaning methods becomes necessary. Chemically enhanced backwash, maintenance cleaning, and intensive chemical cleaning are some chemical cleaning methods that are used in addition to the physical cleaning strategies. Intensive cleaning is recommended at high TMP values where further filtration is no longer sustainable. The main cleaning agents used for MBR fouling are sodium hydroxide (NaOH), sodium hypochlorite (NaOCl) for organic foulants, acids such as citric acid, nitric acid, hydrochloric acid for inorganics and other agents such as ethylene diamine tetra acetic acid (EDTA) or ozone or CO_2_ purging [76,142,143]. Organic molecules present in biofilm could be hydrolyzed and loosen by NaOCl. Chemically enhanced backwash is a daily method that could be applied using chemical agents under normal conditions, while maintenance and intensive cleanings are recommended to be used weekly and once/twice a year, respectively. For a complete cycle, a moderate reagent concentrations of cleaning agents (0.01 wt. % NaOCl) could be used for about 30 min in each cycle, while higher reagent concentrations (0.2–0.5 wt. % NaOCl coupled with 0.2–0.3 wt. % citric acid or 0.5–1 wt. %oxalic acid) are used for intensive cleaning [4,115]. EDTA is effective in removing inorganics present on the membrane surface. This removal is achieved through the forming of a strong complex with Ca^2+^ by replacement of Ca^2+^ ions of biopolymers by EDTA via a ligand exchange reaction [144].

Recently, sonification and vibration through ultrasonic irradiation received noticeable research interest. Sonification cleaning process could be used for removing cake layers in MBRs by breaking down the foulants into smaller fragments. In comparison to other cleaning methods, this process is less applicable in all MBRs due to the appearance of pore blocking which decreases the efficiency of this method. Sonification is recommended coupled to backwashing and chemical cleaning to achieve almost complete flux recovery. However, this is not applicable in large-scale systems due to the focused nature of the sonic energy [115]. 

Although chemical cleaning methods are efficient on removing fouling and scaling, the frequent application of chemical agents could damage the membrane and shorten the membrane life. This damage is more effective on polymeric membranes widely used in AnMBRs, which are less resistant against chemicals, especially oxidants like chlorine. Therefore, progressive research has been conducted on using ceramic and conductive membranes in AnMBRs, showing low propensity to fouling and remaining stable against chemical cleaning [4,76,142].

#### 5.3.3. Anti-Fouling Membranes

Based on the need of fouling control and clogging in full-scale MBRs to achieve long-term operation, some other methods are applied such as the chemical modification of the mixed liquor and the chemical modification of membrane surface for fouling mitigation. Addition of substances such as coagulants, polyelectrolytes, adsorbing agents and membrane performance enhancers could change the mixed liquor characteristics and enhance membrane efficiency in long-term operations and reduce fouling. Flocculation of biomass could be improved by adding coagulants, with a consequent neutralization of negative charges by introducing positive ions. Adsorbents like natural zeolite and activated carbon could reduce fouling by removing colloidal and soluble compounds [145]. In other researches it is reported that adding sponge and powdered activated carbon could reduce cake formation and causes fouling mitigation [103]. Other additives like the cationic polymer MPE50 and poly-aluminum chloride are also effective in controlling membrane fouling. However, besides the effectiveness of using additives, economical aspects of this method should be considered in order to attain surety on the implications and cost-effectiveness of this strategy in full-scale MBRs [103]. 

Chemical modification of the membrane’s surface could also be employed to improve the membrane performance in long-term applications. Polymerisable bicontinuous microemulsion (PBM) technique was used to prepare a surface modified antifouling UF membrane. The developed novel membrane was much more resistant to fouling compared to conventional MBRs. Recently, application of engineered nanomaterials in different fields received more attention due to their unique properties. Graphene, graphene oxide, carbon nanotubes (CNTs), fullerenes, silver (Ag), titanium dioxide (TiO_2_), and zinc oxide (ZnO) are some of the nanomaterials used in MBR systems due to the outstanding properties of hydrophilicity antimicrobial ability and photocatalytic activity. These unique properties may result in controlling and mitigation of membrane fouling [62,142]. In the research conducted by Zhao et al. [103], a polyvinylidene fluoride/ hydrophilic graphene oxide nanosheets (PVDF/ GO) membrane was developed, which exhibited lower membrane resistance and cleaning frequency compared to a conventional PVDF membranes [103]. Antimicrobial activity of Ag nanoparticles makes them a suitable material to control membrane fouling, by preventing attachment of microorganisms to the membrane surface. The unique problem in using these nanoparticles is dissolution of Ag ions in the liquid medium, which restricts its application [62,146,147,148]. ZnO and TiO_2_ nanoparticles could enhance also the membrane fouling resistance by increasing the membrane hydrophilicity and their antimicrobial ability [148,149,150,151]. Application of TiO_2_ nanoparticles is also limited due to toxicity of these particles on microorganisms present in bioreactors [62,152,153]. CNTs and graphene-based nanomaterials were also studied in some researches dealing with by their incorporation into the membranes, resulting in an improved permeability and simplified fouling control due to their unique properties, resulting in long-term operations. It should be noted that the utilization of CNTs in membranes is considered to be quite economical for large-scale applications in MBRs since they are insoluble in water [154,155].

The integration of advanced oxidation processes with MBRs or electrocoagulation with MBRs, the integration of microbial fuel cells to MBRs (MFC-MBR), improving aeration system in MBRs to reduce concentration polarization, increasing turbulent shear stress, mechanical cleaning of membrane fouling by equipping MBRs with biofilm carriers (BCs), application of shear-enhanced membranes such as rotating and vibrating membranes and using rotating annular membrane filters are some of the strategies recommended in different studies, ensuring high levels of fouling mitigation in MBRs/AnMBRs [115,156].

## 6. Biohydrogen Separation and Purification

Since biohydrogen is produced along with other gases such as CO, CO_2_, N_2_, and CH_4_ [157,158], it is necessary to study the biohydrogen separation technologies. The efficiency and selectivity of the membrane used for hydrogen separation is completely dependent on the membrane material. Organic (polymer or carbon) and inorganic (metallic or ceramic) membranes are used in different studies for hydrogen separation. Despite the high selectivity and remarkable efficiency of metallic membranes in hydrogen separation, several limitations such as cost and mechanical stability restrict the application of this type of membranes [159,160]. Therefore, polymeric membranes are preferred as an interesting technology for hydrogen separation due to their low cost and high stability against high pressure drops [157].

### H_2_ Selective Membranes and Operational Conditions

Numerous studies have been conducted on finding suitable polymeric membranes for hydrogen purification regarding efficiency and cost-effectiveness. Membranes made of glassy and rubbery polymers are usually recommended for this purpose based on their permeability and selectivity properties. Differences in diffusivity is the base of separation in H_2_-selective glassy membranes, while separation occurs based on solubility differences of the permeating molecules in CO_2_-selective rubbery membranes. Rubbery polymeric membranes possess high permeability with a relatively low selectivity, while membranes made of glassy polymers are reported to have high selectivity and lower permeability. The choice of the proper membrane is also dependent on the composition of the gas mixture. The membrane separation efficiency for gas pairs is usually better than gas mixture, and this is due to the interactions present among gas molecules and the polymer matrix, changing the permeation manner of the individual gas types. However, different separation limiting phenomena like concentration polarization, competitive sorption and mass transfer could appear in multi-component gas mixtures separation [41,157,161].

For gas pairs, the polymeric membranes used separating H_2_ are categorized as H_2_-selective/CO_2_-rejective and CO_2_-selective/H_2_-rejective ones. Penetration of gases through these membranes could be described by the solution-diffusion model, which is based on diffusivity and solubility differences of the present components. The main permeation driving force is represented by the hydrogen partial pressure difference across the membrane. Glassy polymers sieving molecules are commonly used in H_2_-selective membranes in which H_2_ separation is based on size and permeating priority of H_2_ over other substances e.g., CO_2_. CO_2_-selective/H_2_-rejective membranes normally work contrariwise of the above mentioned membranes and H_2_ is less soluble in these membranes. H_2_/N_2_ separations are simpler than H_2_/CO_2_ since N_2_ transport is significantly slower than hydrogen [162].

In gas mixtures, the importance of gas composition is proved by the results reported for different membranes in the literature. Different PEBA/PEG blend membranes are employed for both gas mixture (H_2_, CO_2_, N_2_) and gas pair (H/CO_2_) by Car et al. [163]. It was evidenced that the efficiency of the CO_2_-selective membrane was remarkably better for gas pair compared to gas mixture, regardless of the applied polymer composition. Findings of the report conducted by Reijerkerk et al. on the application of PEBAX/PEGPDMS membranes and the study done by Yave et al., who used poly(amide-b-ethylene oxide)/polyethylene glycol blend membranes, are also in agreement with the mentioned results [164,165]. Comprehensive study were done by David et al. on polyimide (Matrimid) membranes using binary H_2_/CO_2_ and ternary H_2_/CO_2_/N_2_ mixtures, showing that the H_2_/CO_2_ separation factor is significantly dependent on the experimental conditions [166]. The results exhibited that H_2_ selectivity is independent of nitrogen, but it is highly affected by carbon dioxide concentration [41]. Recently, the ionic liquid (IL) based membranes were introduced, as a result of the combination of porous polymeric membranes and ionic liquids, leading to the preparation of nonporous membranes, named as supported ionic liquid membrane (SILM). They exhibit high selectivity for the separation of H_2_, CO_2_, and N_2_, as well [41,167].

Biohydrogen separation employing polymeric membranes is also influenced by the operating conditions applied in the AnMBR. Feed pressure, temperature, and stage cut are some of these parameters, which should be optimized to reach the maximum biohydrogen separation efficiency. Hydrogen partial pressure difference between feed and permeate side plays an important role as a driving force affecting the process efficiency. It is important to keep the system at high values of H_2_ partial pressure difference to achieve high flux and selectivity in AnMBRs. This is possible by applying vacuum on the permeate side in lab-scale system, not applicable in full-scale MBRs due to economical feature. Temperature is the most effective factor, which should be kept at moderate or slightly elevated values for H_2_-selective membranes, while lower temperatures are needed for CO_2_-selective membranes. Consequently, various parameters and operational conditions such as temperature and pressure control, undesirable biofilm formation on the surface of the separation membrane, moisture content, and fermentation control must be accomplished to optimize the process and reach the maximum process efficiency [41,168].

## 7. Conclusions

The present review emphasized the potential of biohydrogen production in MBRs as integrated systems. Biohydrogen production from municipal waste and wastewater via anaerobic fermentation is a cost-effective process, which may make hydrogen as the sustainable and efficient energy carrier of the future due to its characteristics of cleanliness, high energy density and abundance, and environment-friendly generation method. Direct and indirect biophotolysis, photo-fermentations, and dark fermentation are introduced and discussed as the common technologies for biohydrogen production, nevertheless still at research and development stage. Improving biohydrogen production systems requires a comprehensive study on the suitable hydrogen producing bacteria and metabolic engineering, optimum bioreactor design, operational fermentation conditions (e.g., pH and temperature), using proper membranes for rapid removal and purification of H_2_. Besides the several advantages of biohydrogen production in MBRs, the relatively low biohydrogen production rate, yield, practicality and economic feasibility of biohydrogen production in AnMBRs, membrane fouling and scaling up are the major challenges remaining to be overcome before a feasible practical process to achieve better performance and a more predictable, controllable and long-term steady-state operation.

## Figures and Tables

**Table 1:** Dark fermentation bioreactors used for H_2_ production with different substrates.

Microorganisms	Substrate	Type of Reactor	H_2_ Rate (l H_2_/l/h)	Reference
Sludge (wastewater treatment plant)	Molasses	CSTR	0.20	[48]
Sludge (wastewater treatment plant)	Glucose	ASBR	0.23	[49]
Sludge (wastewater treatment plant)	Sucrose	FBBAC	1.2	[50]
Activated sludge and digested sludge	Glucose	AFBR	2.4	[51]
Sludge (wastewater treatment plant)	Sucrose	UASB	0.27	[52]
Sludge (wastewater treatment plant)	Sucrose	CIGSB	9.3	[53]
Sludge (wastewater treatment plant)	Sucrose	FBR	1.4	[54]
Sludge (wastewater treatment plant)	Glucose	AFBR	7.6 biofilm; 6.6 granules	[55]
Heat-treated soil	Glucose	MBR	0.38	[56]
Heat shock treated anaerobic sludge	Food waste	LBR	0.15	[57]
Sludge (wastewater treatment plant)	Vegetable kitchen waste	ICSTR	0.04	[58]
Adapted anaerobic sludge	Cheese whey	Batch	0.003	[59]

**Table 2:** Membrane properties of different ThAeMBRs.

Type of Wastewater	Type of MBR	Membrane Characteristics					Configuration	Flux (LMH)	Ref.
		Membrane Process	Module	Pore Size (μm)	Area (m^2^)	Material			
Molasses-based synthetic wastewater	ThAeMBRs	MF/UF	Submerged	0.45 μm/ 150 kDa	0.0125	Filtanium Ceramic	Hollow fiber	48/72	[64]
TMP pressate	ThAeMBRs	MF	Submerged	0.3 μm	0.03	PVDF	Flat sheet	6.8–11.8	[65]
Paper drinking wastewater	ThAeMBRs	UF	Submerged	0.04 μm	0.34	PVDF	Flat sheet	6–25	[66]
Pharmaceutical wastewater	ThAeMBRs	UF	External	300 kDa	N.S.	Ceramic	Tubular	N.S.	[67]
Sewage sludge	ThAeMBRs	UF	External	10 nm	N.S.	Ceramic	N.S.	N.S.	[68]
Industrial liquid wastes	ThAeMBRs	UF	External	300 kDa	N.S.	Ceramic	Tubular	N.S.	[69]
Sewage sludge	ThAnMBRs	UF	External	300 kDa	0.0226	TiO_2_/ ZrO_2_ Ceramic	Tubular	7	[70]
Synthetic molasses	ThAnMBRs	UF	Submerged	10 kDa	0.1	polysulphone	Tubular	6	[71]
Prehydrolysis Liquor	ThAnMBRs	MF	Submerged	0.4 μm	0.11	Chlorinated polyethylene	Flat sheet	4	[72]
Glucose model solution	ThAnMBRs	N.S.	External	40 nm	~0.033	Ceramic (α-Al_2_O_3_)	Hollow fiber	40.3–72.2	[73]

N.S.: not specified.

**Table 3:** Bioydrogen production efficiency of different AnMBRs.

Inoculum	Substrate	Retention Time		H_2_ Generation Performance		Ref.
		Hydraulic	Solid/Biomass	Yield	Productivity	
Heat-treated soil inocula	Glucose	3.3–5	3.3–48 h	N.S.	9.2 L H_2_/L-d	[56]
Acid-treated, acclimated sludge	3 Hexoses	1–4 h	N.S.	39 L H_2_/mol glucose	66 L H_2_/L-d*	[89]
Anaerobic sludge	Glucose	4 h	N.S.	38.1 L H_2_/mol glucose	25 L H_2_/L-d	[89]
Heat-treated sludge	Glucose	9 h	450 d	N.S.	2.5 L H_2_/L-d	[80]
Screened anaerobic digester sludge	Glucose	8 h	24 h	40.2 L H_2_/mol glucose	4.5 L H_2_/L-d	[90]
Heat-treated sludge	Glucose	9 h	12.5 h	35.4 L H_2_/mol glucose	5.9 L H_2_/L-d	[81]
Heat-treated, acclimated sludge	Glucose	N.S.	90 d	19.5 L H_2_/mol glucose	2.5 L H_2_/L-d	[85]
Screened anaerobic digester sludge	Glucose	8 h	24 h	40.3 L H_2_/mol glucose	4.5 L H_2_/L-d	[90]
Sludge	Glucose	9 h	90 d	19.2 L H_2_/mol glucose	2.56 L H_2_/L-d	[85]
Heat-treated, acclimated sludge	Glucose	9 h	2–90 d	27 L H_2_/mol glucose	5.8 L H_2_/L-d	[91]
Acclimated sludge	Glucose	8 h	24 h	N.S.	4.4 L H_2_/L-d	[82]
Heat-treated sludge	TPW	2–8 h	N.S.	42.4 L H_2_/mol hexose**	19.8 L H_2_/L-d	[92]
Sludge	Waste bread	6 h	N.S.	0.109 L H_2_/mol waste bread	7.4 L H_2_/L-d	[93]
Anaerobic granular sludge	Glucose	4 h	N.S.	44.8 L H_2_/mol glucose	11.4 L H_2_/L-d	[94]

N.S.: not specified; TPW: Tofu processing waste; *: on fructose; **: hexose added.

**Table 4:** Different fouling types.

Definition	Fouling Rate (mbar/min)	Time Interval	Cleaning Method Applied
Reversible/temporary fouling	0.1–1	10 min	Physical cleaning
Residual fouling	0.01–0.1	1–2 week	Maintenance cleaning (e.g., chemically enhanced backflush)
Irreversible/permanent fouling	0.001–0.01	6–12 months	Chemical cleaning
Irrecoverable fouling	0.0001–0.001	Several years	Cannot be removed

## List of Acronyms

AD	Aanaerobic digestion
AeMBR	Aerobic membrane bioreactor
AFBR	Anaerobic fluidized-bed reactor
AnMBR	Anaerobic membrane bioreactor
ASBR	Anaerobic Sequencing Batch Reactor
ATP	Adenosine triphosphate
CIGSB	Carrier-induced granular sludge bed reactor
CNT	Carbon nanotubes
COD	Chemical oxygen demand
CSTR	Continuous stirred tank reactor
EPS	Extracellular polymeric substances
FBBAC	Fixed bed bioreactor with activated carbon
FBR	Fixed bed reactor
FHL	Formate hydrogenlyase
FS	Flat sheet
GO	Graphene oxide
HF	Hollow fiber
HRT	High retention time
ICSTR	Intermittent continuous stirred tank reactor
LBR	Leaching bed reactor
MBR	Membrane bioreactor
MF	Microfiltration
MFC	Microbial fuel cells
MLSS	Mixed liquor suspended solid
MT	Multi tubular
NADH	Nicotinamide adenine dinucleotide
NF	Nanofiltration
OLR	Organic loading rate
PE	Polyethylene
PES	Polyethersulfone
PFL	Pyruvate formate lyase
PFOR	Pyruvate ferredoxin oxido reductase
PNS	Photosynthetic non-sulfur
PP	Polypropylene
PS	Polysulfone
PVDF	Polyvinylidene difluoride
SMP	Soluble microbial products
SRT	Solid retention time
TCA	Tricarboxylic acid cycle
ThAeMBR	Thermophilic aerobic membrane bioreactor
ThAnMBR	Thermophilic anaerobic membrane bioreactor
TMP	Transmembrane pressure
UASB	Up-flow Anaerobic Sludge Blanket
UF	Ultrafiltration
VFA	Volatil fatty acid
